# Brain responses to the vicarious facilitation of pain by facial expressions of pain and fear

**DOI:** 10.1093/scan/nsac056

**Published:** 2022-10-06

**Authors:** Ali Khatibi, Mathieu Roy, Jen-I Chen, Louis-Nascan Gill, Mathieu Piche, Pierre Rainville

**Affiliations:** Centre of Precision Rehabilitation for Spinal Pain (CPR Spine), School of Sport, Exercise and Rehabilitation Sciences, College of Life and Environmental Sciences, University of Birmingham, Birmingham B15 2TT, UK; Centre for Human Brain Health, University of Birmingham, Birmingham B15 2TT, UK; Research Centre of the Institut Universitaire de Gériatrie de Montréal, Université de Montréal, Montréal, QC H3W 1W5, Canada; Research Centre of the Institut Universitaire de Gériatrie de Montréal, Université de Montréal, Montréal, QC H3W 1W5, Canada; Department of Psychology, McGill University, Montréal, QC H3A 1G1, Canada; Alan Edwards Centre for Research on Pain, McGill University, Montreal, QC H3A 0G1, Canada; Research Centre of the Institut Universitaire de Gériatrie de Montréal, Université de Montréal, Montréal, QC H3W 1W5, Canada; Department of Stomatology, Université de Montréal, Montréal, QC H3T 1J4, Canada; Research Centre of the Institut Universitaire de Gériatrie de Montréal, Université de Montréal, Montréal, QC H3W 1W5, Canada; Department of Anatomy, Université du Québec à Trois-Rivières, Trois-Rivières, QC G8Z 4M3, Canada; Research Centre of the Institut Universitaire de Gériatrie de Montréal, Université de Montréal, Montréal, QC H3W 1W5, Canada; Department of Stomatology, Université de Montréal, Montréal, QC H3T 1J4, Canada

**Keywords:** pain, vicarious facilitation, nociceptive flexion reflex, fMRI, emotional facial expression

## Abstract

Observing pain in others facilitates self-pain in the observer. Vicarious pain facilitation mechanisms are poorly understood. We scanned 21 subjects while they observed pain, fear and neutral dynamic facial expressions. In 33% of the trials, a noxious electrical stimulus was delivered. The nociceptive flexion reflex (NFR) and pain ratings were recorded. Both pain and fear expressions increased self-pain ratings (fear > pain) and the NFR amplitude. Enhanced response to self-pain following pain and fear observation involves brain regions including the insula (INS) (pain > fear in anterior part), amygdala, mid-cingulate cortex (MCC), paracentral lobule, precuneus, supplementary motor area and pre-central gyrus. These results are consistent with the motivational priming account where vicarious pain facilitation involves a global enhancement of pain-related responses by negatively valenced stimuli. However, a psychophysiological interaction analysis centered on the left INS revealed increased functional connectivity with the aMCC in response to the painful stimulus following pain observation compared to fear. The opposite connectivity pattern (fear > pain) was observed in the fusiform gyrus, cerebellum (I–IV), lingual gyrus and thalamus, suggesting that pain and fear expressions influence pain-evoked brain responses differentially. Distinctive connectivity patterns demonstrate a stronger effect of pain observation in the cingulo-insular network, which may reflect partly overlapping networks underlying the representation of pain in self and others.

## Introduction

Facial expressions are the main channel for the communication of emotions in social settings. Observation of emotions informs the observer about the observed person’s condition and influences the observer’s reaction and processing of information (Hadjistavropoulos *et al.*, 2011). Pain observation has been shown to be associated with improved action readiness, facilitated spinal nociceptive processing and heightened pain perception in the observer (Avenanti *et al.*, 2006; Mailhot *et al.*, 2012; Khatibi *et al.*, 2015). However, other studies suggest that such facilitation is not specific to pain communication and reflects the negative valence of pain expressions (e.g. Wiech and Tracey, 2009; Bayet *et al.*, 2014).

There are several proposals about the mechanisms underlying the vicarious facilitation of pain. The perception–action model predicts that mapping the observed emotional state into a similar network in the brain of the observer enables the observer to understand the observed person and to prepare appropriate reactions (Preston and de Waal, 2002). The model was developed to explain the perception of motor actions by observing them in others. Discovering mirror neurons provided physiological evidence supporting this hypothesis (Iacoboni *et al.*, 1999). Observing shared representations of firsthand and observed emotions in the brain further extended this model (Preston, 2007). Some studies show that observation of pain is associated with activation in brain areas [such as the anterior INS and the anterior cingulate cortex (ACC)] involved in self-pain perception (Budell *et al.*, 2010; Jauniaux *et al.*, 2019). These findings, along with the observed activation in areas such as the inferior parietal lobule (IPL) and inferior frontal gyrus (IFG), which are parts of the so-called ‘human mirror neuron system’, support the perception–action model on the role of a sensory-motor resonance in the facilitation of responses to pain (Budell *et al.*, 2015). On the other hand, the motivational priming theory predicts the facilitation of responses to negative stimuli as a result of the activation of the defensive system (Williams and Rhudy, 2012). Accordingly, the output of brain networks involved in arousal (Kragel and LaBar, 2016) may facilitate the activity of the pain-processing network when viewing any negative expression. Based on the assumptions of this model, the brain mechanisms underlying the facilitation of pain following the observation of others’ pain expressions are not specific to pain and might be triggered similarly by other negative expressions.

This pain facilitation may be detected at the spinal and supraspinal levels of the neuraxis. The nociceptive flexion reflex (NFR) is suggested to be indicative of the spinal processing of nociceptive signals (Sandrini *et al.*, 2005). Previous studies showed that the NFR is facilitated following the observation of pain in others (Vachon-Presseau *et al.*, 2011; Mailhot *et al.*, 2012). But increased pain and NFR are also reported following other negatively valenced stimuli (Rhudy *et al.*, 2005; Roy *et al.*, 2009), consistent with the motivational priming theory. To our knowledge, no brain imaging study has examined directly if pain facilitation produced by pain observation or negative emotions involves distinctive processes.

A neuroimaging study examining brain correlates of vicarious facilitation of pain during observation of pain and a comparable arousing negative emotion would (i) allow testing the specificity hypothesis on spinal and supraspinal facilitation of pain responses and (ii) further test the neural correlates of vicarious pain facilitation. Based on the motivational priming hypothesis, we expect to see (i) increased NFR amplitude following observation of both pain and negative (here fear) expressions and (ii) comparable increased activation in the frontotemporal network when participants observe pain or fear. On the other hand, the sensorimotor resonance hypothesis predicts (i) increased NFR amplitude to pain observation greater than fear and (ii) increased pain response in the pain network following observation of pain greater than following observation of fear expression.

## Methods

### Participants

A total of 22 participants were recruited through advertisements on local community websites. One participant was excluded before testing for medical reasons. Finally, 21 pain-free healthy volunteers were tested and were included in this study (10 females and 11 males; mean age = 25.24, SD = 4.13, range: 19–32). This sample size was determined based on the results of our previous studies showing the facilitation of pain, the NFR or brain response to facial expressions of pain (Budell *et al.*, 2010, 2015; Vachon-Presseau *et al.*, 2011, 2012; Mailhot *et al.*, 2012; Khatibi *et al.*, 2014). All subjects read and signed a printed copy of informed consent approved by the research ethics committee of the Centre de Recherche de l’Institut Universitaire de Gériatrie de Montréal (CRIUGM) in Montréal, Canada (CMER-RNQ 12-13-018).

### Electrocutaneous stimulation and the NFR

The NFR was elicited by transcutaneous electrical stimulation of the right sural nerve on its retromalleolar path using two 1 cm surface electrodes. The delivery of electrical stimuli was controlled by a computer running E-Prime 2.0 software (Psychology Software Tools, Inc., Sharpsburg, PA, USA) connected to a Grass stimulator (Model S48, Grass Technologies, West Warwick, RI, USA) driving an optically isolated constant current stimulator (DS7A, Digitimer, Welwyn Garden City, England). Electromyographic (EMG) activity was recorded from the short head of the right biceps femoris muscle using two surface electrodes (EL503, BIOPAC Systems, Inc., Goleta, CA, USA). The ground electrode was placed on the medial aspect of the tibial tuberosity. The skin was previously degreased, shaved and abraded in order to reach an impedance <10kΩ between the ground and each of the two other electrodes. EMG activity was amplified 2000 times, filtered (10–500 Hz), sampled at 10 000 Hz (MP150; BIOPAC Systems, Inc., Goleta, CA, USA) and stored for offline analysis on a computer running AcqKnowledge 4.0. (BIOPAC Systems, Inc., Goleta, CA, USA).

The NFR threshold was determined individually using the staircase method by increasing and decreasing the stimulus intensity in 1.0 and 0.5 mA steps (at least three stimuli per intensity and at least 6 s between two consecutive stimuli) (Khatibi *et al.*, 2014). The NFR threshold was defined as the lowest stimulus intensity evoking a stable response (i.e. a clearly detectable EMG burst in more than 80% of the trials), adapted from the classic work of Willer (Willer, 1977; Piche *et al.*, 2009, 2010). Two levels of shock intensity were used in this study (high and low). For the high-intensity (noxious) shock, the stimulus intensity was adjusted to 120% of the NFR threshold. The mean (±SD) stimulus intensity (120% threshold) for the 20 participants was 6.7 mA (±2.5; range 3.5–12). The mean rating of pain intensity was 43.1 (±6.7; range 35–55/100). For the low-intensity shock, the intensity was set at 50% of the high intensity (3.36 ± 1.26) and evoked no NFR. For low shock, the mean rating was 15.01 (±14.0; range 0–39/100). This stimulus was included in the paradigm to introduce some uncertainty regarding pain sensation, but it was not considered further in the analysis of pain modulation by facial expressions.

### Facial expressions

Twenty-four video clips of 1-s dynamic facial expressions from eight actors (four females) were selected from a validated database (Simon *et al.*, 2008; Roy *et al.*, 2007). The selected pain expression clips were the same as those used in our previous brain imaging studies (Simon *et al.*, 2006, 2008; Budell *et al.*, 2010, 2015). These video clips were rated for arousal and valence by an independent group of participants (*n* = 20). Three video clips were taken from each actor expressing the following expressions: neutral, pain and fear. Pain and fear expressions were selected to match arousal ratings based on pre-experimental testing. Thus, pain expressions with lower intensity and valence ratings were selected to match the arousal with the fear expressions (arousal pain expressions = 5.10 ± 1.1, arousal fear expressions 4.93 ± 1.4 and valence pain expressions = 3.17 ± 0.6, valence fear expressions = 3.86 ± 0.6). Videos were presented in black and white over a gray background.

### Procedure

**Fig. 1.**
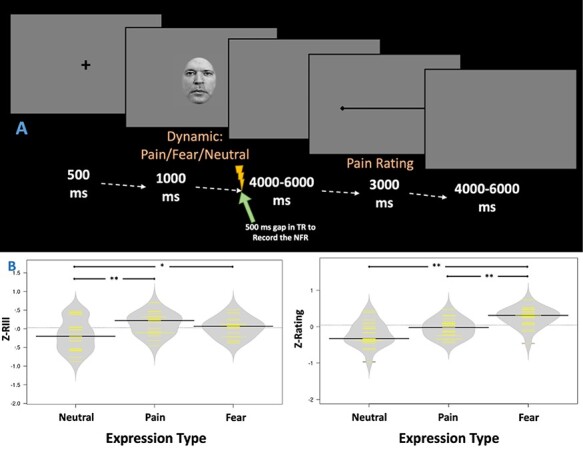
(A) Trials started with a 500 ms fixation point, followed by a 1-s clip displaying a pain, fear or neutral expression and ending with the application of a high-intensity shock in 33% of the trials. The timing of each trial was synchronized with the fMRI acquisition to allow for the measurement of the NFR during a 500 ms gap introduced between volumes (see details in fMRI image acquisition and analyses). The electrical stimulus was followed by a 4000–6000 ms delay before subjects rated the pain produced by the electrical stimulation. The total duration of a trial was 15 000 ms. (B) Graphs show the magnitude of individually Z-standardized NFR (left panel), and pain ratings (right panel) elicited by the high-intensity shock administered after clips displaying neutral, pain or fear expressions. Each yellow line represents an individual subject, and the black bars represent the group means. **P < 0.001, *P < 0.01.

A computer running the E-Prime 2.0 software (Psychology Software Tools, Inc., Sharpsburg, PA, USA) controlled the presentation of the facial expressions, the administration of the electrical stimulation and the VAS. Before the start of the experimental runs, a shock-only run (localizer run) with six high-intensity shocks and six low-intensity shocks was carried out. This run was followed by two experimental runs, as described below. Figure 1A presents an overview of a trial in the experimental runs. Each trial started with a fixation point at the center of the screen (500 ms), followed by a dynamic facial expression (1000 ms). At the end of the presentation of the facial expression, a high-intensity shock was delivered in 33% of trials for which the NFR was recorded (500 ms time window). This was followed by a gap of 4000–6000 ms, after which participants were asked to rate their pain on a VAS (from 0 = not painful at all to 100 = extremely painful). After 3000 ms, the VAS disappeared, and the next trial started after a delay of 4000–6000 ms. The total duration of a trial was 15 000 ms. There were two runs with a gap in between (after 1 min, the subject was asked if she/he was ready, and then the second run was started). Each run had 36 trials (33% high-intensity shocks, 33% low-intensity shocks and 33% no shocks), and the total duration of the run was about 9 min.

### Physiological and behavioral data analysis

To determine NFR amplitude, EMG activity was full-wave rectified and the integral value of this signal was calculated for the 90–180 ms post-stimulus time window for each shock. The NFR amplitude and pain ratings were z-transformed individually across trials. Mean Z-Score for each type of expression were calculated and used in the analysis. A regression analysis with Z-Score as the dependent variable and the type of expression (dummy coded) as the predictor was used to compare the effect of the type of expression on pain ratings and NFR. Point estimate Cohen’s *d* is presented as the estimation of the effect size in paired sample comparisons.

### MRI image acquisition and analyses

Imaging data were acquired using a 3.0T whole-body scanner (Siemens Trio TIM) with a 32-channel head coil at the Unié de Neuroimagerie Fonctionnelle (UNF) of the CRIUGM. Blood oxygenation level-dependent (BOLD) signal was acquired using a T2*-weighted simultaneous-multi-slice (SMS) EPI sequence (simultaneous excitation = 3 slices, TR = 2000 ms, TR delay 500 ms, TE = 20 ms; flip angle = 70°; matrix size = 74 × 74; FOV = 220 × 220 mm^2^; 255/110 volumes; 51 interleaved, AC–PC axial slices per whole-brain volume at 3 mm thickness; in-plane resolution= 2.97 × 2.97 mm^2^; parallel imaging with GRAPPA 2; bandwidth= 1732 Hz/Px). The TR delay was included to enable us to record noise-free EMG activity to measure the NFR. Structural images were acquired using a high-resolution, T1-weighted multiecho MPRAGE sequence (TR = 2530 ms; TE = 1.64, 3.50 and 5.36, flip angle = 7°; FOV = 256 mm; matrix size = 256 × 256; 1 mm isotropic resolution; 176 slices per whole-brain volume; parallel imaging with GRAPPA 2; bandwidth = 651 Hz/Px).

Data preprocessing and analysis were done using FSL-5.0 and in-house MATLAB codes. Steps for the offline preprocessing of functional images are described below.

#### Brain T1 anatomical processing

Anatomical images were skull-stripped using the brain extraction tool (BET) implemented in FSL. Cerebral spinal fluid (CSF) and white matter (WM) masks were created for each subject and registered to the fMRI data for the extraction of signal time series, which were subsequently used as a covariate of no interest in the functional analysis as described below.

#### Brain fMRI preprocessing

Functional images were skull-stripped using BET implemented in FSL. The first two volumes were removed. Motion correction was conducted using MCFLIRT (Jenkinson *et al.*, 2002) before applying the high-pass temporal filter (100 Hz) and a Gaussian kernel of 5 mm for spatial smoothing. Functional data were then co-registered to the high-resolution T1 anatomical image and transformed into the MNI space (MNI-152-2 mm) using FLIRT to allow for group analysis (Jenkinson and Smith, 2001; Jenkinson *et al.*, 2002). The FNIRT nonlinear registration (Andersson *et al.*, 2007a,b) was used to further refine the registration.

#### Brain fMRI analysis

The FEAT in the FSL was used for the analysis. Time-series statistical analysis was carried out using FILM with local autocorrelation correction (Woolrich *et al.*, 2001). An event-related model was used for the analysis of subject-level data. A gamma function with temporal derivatives was used to model the HRF. Motion correction parameters (six), the mean CSF signal and the mean WM signal were included as confounds. The shock-only run data was analyzed separately to obtain a reference map of shock-pain activation independent from the presentation of facial expression. In experimental runs, each type of facial expression (pain *vs* fear *vs* neutral) was entered as a separate regressor coding for the facial stimuli not followed by shocks. For the trials where a high-intensity shock (33% of trials) followed the presentation of the facial expression, only the shock was modeled because of the strong collinearity between face stimuli and shocks imposed by the experimental paradigm. One regressor was created for the shocks following each type of expression. This allowed examining brain responses to facial expressions alone, high-intensity shocks alone and high-intensity shocks following the three facial expressions.

A mid-level mixed general linear model (GLM) was used for the contrasts, and the group-level fixed-effect model was carried out using FLAME12. All group-level statistical maps were corrected for multiple comparisons using the Gaussian Random Field (GRF) theory [*Z*(cluster forming) > 2.3, *P*(cluster-corrected)  < 0.05 (Worsley *et al.*, 2002)].

A psychophysiological interaction (PPI) analysis (Friston *et al.*, 1997) was performed to examine changes in functional connectivity of a designated brain region activated by noxious shocks across conditions. A new GLM was constructed at the individual level using the previously described regressors to model the main effect of the condition (psychological regressors in the PPI model; three regressors: shock following observation of neutral, pain and fear expressions) and the mean signal in the selected ROI (physiological regressor; left INS: higher activation in response to shocks following observation of pain and fear faces than the observation of neutral faces, XYZ = −35, 0, 6 and 10 mm diameter). The last three regressors represented the interactions between the psychological regressors and the physiological regressor (PPI). All three psychological regressors were convolved with the HRF, and the design matrix included movement parameters (six regressors), mean CSF and mean WM signal (two regressors). A GRF correction was applied to the group-level analysis. A significant PPI indicates a change in the functional connectivity between the designated brain region (here the insular cortex) and the reported brain regions related to the specific condition. Contrasts were run to reveal changes in the pattern of connectivity associated with shock-evoked responses following the pain and fear *vs* neutral faces, pain alone *vs* neutral, fear alone *vs* neutral and pain *vs* fear.

## Results

### Behavioral and EMG responses

A multilevel regression analysis with the magnitude of RIII (*Z*-Score, within-subject; Figure 1B, Left) as the dependent variable and faces (fear *vs*. pain *vs*. neutral) as the predictors was run. The model could explain 14% of variance [*F*(2,62) = 4.87; *P* = 0.01]. It showed that both painful (Raw = 5.81 ± 4.9 mV; *Z*-Score = 0.13 ± 0.3; Beta = 0.37; *t* = 2.65, *P* = 0.01) and fearful expressions (Raw = 5.46 ± 4.7 mV; *Z*-Score = 0.04 ±0.2; Beta = 0.38; *t* = 2.75, *P* = 0.008) increased NFR amplitude significantly relative to the neutral faces (Raw = 5.10 ± 4.3 mV; *Z*-Score = −0.17 ± 0.4). The paired comparison revealed no significant difference in NFR amplitude following the observation of pain compared with fear (*P* = 0.24, Cohen’s *d* = 0.262). When the raw scores were included instead of the z-transformed scores, the pattern of results and level of significance remained unchanged.

A similar analysis of pain ratings following high-intensity shock (*Z*-Score, within-subject; Figure 1B, Right) revealed that the regression model could explain 31% of the variance [*F*(2,62) = 13.64; *P* < 0.001]. It showed that fearful expressions could explain the variance in pain ratings (Beta = 0.64; *t* = 5.16, *P* < 0.001), but for painful expressions, it only showed a trend toward significance (Beta = 0.23; *t* = 1.87, *P* = 0.07). Paired sample comparisons revealed that pain ratings following the observation of fear (Raw = 41.14 ± 17.4; *Z*-Score = 0.25 ± 0.3) were higher than those following the observation of pain (Raw = 39.04 ± 16.7; *Z*-Score = −0.04 ± 0.3; *P* = 0.001, Cohen’s *d* = 0.751) or neutral expressions (Raw = 37.85.46 ± 17.3; *Z*-Score = −0.21 ± 0.3; *P* = 0.003, Cohen’s *d* = 0.827). When the raw scores were included instead of the z-transformed scores, the pattern of results and level of significance remained unchanged. For the low-intensity shock, observation of pain or fear faces did not result in significant changes in rating.

### Brain responses

Brain responses to facial expressions not followed by shocks were examined first. Then, brain responses were analyzed to examine the impact of facial expression on shock-evoked activity and to test possible differences in the connectivity patterns in these responses.

#### Effects of facial expression

**Fig. 2.**
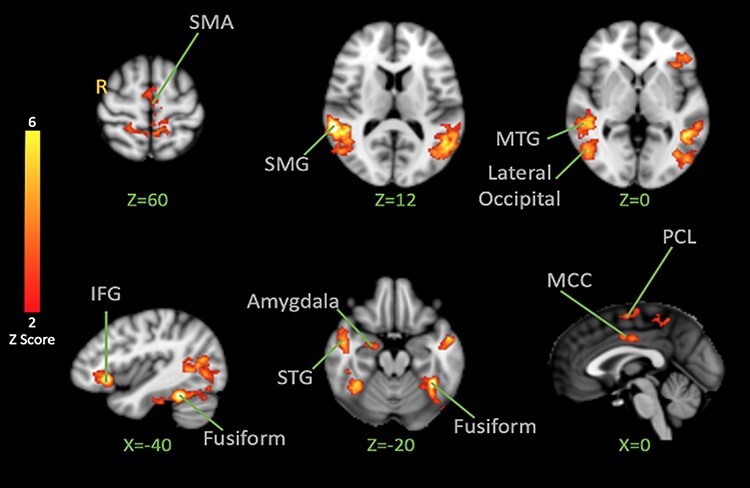
BOLD activation to pain and fear > neutral Facial Expression Observation. The color bar indicates Z-Score. The cluster-based threshold was corrected for multiple comparisons using GRF (*P < 0.05*). Peak coordinates are reported in Table 1.

To understand the brain correlates of vicarious pain facilitation, we first examined responses to pain and fear faces not followed by shocks. Pain and fear observation conditions were tested first together against the observation of neutral expressions (Figure 2). Significant activation was found in the supplementary motor area (SMA), bilateral supramarginal gyrus (SMG), bilateral occipital cortex, the mid-cingulate cortex (MCC), the paracentral lobule (PCL), amygdala, bilateral superior temporal gyrus (STG) and bilateral fusiform (Table 1). These patterns reflect basic brain responses to dynamic facial expressions.

**Table 1.** Brain activation in response to the observation of pain and fear facial expressions *vs* neutral expression

*Z*-value	*X*	*Y*	*Z*	Anatomical label
6.98	48	−42	8	Right superior temporal gyrus
6.39	−38	−48	−22	Left fusiform gyrus
5.88	−50	−56	8	Left superior temporal gyrus
5.70	−40	26	−6	Left inferior frontal gyrus
5.25	38	−46	−24	Right fusiform gyrus
4.77	−54	0	−18	Left middle temporal gyrus
4.27	−8	−42	50	Left paracentral lobule
3.91	−3	−6	60	Supplementary motor area
3.52	−2	−4	38	Cingulate gyrus
3.11	23	−5	−20	Right amygdala

**Fig. 3.**
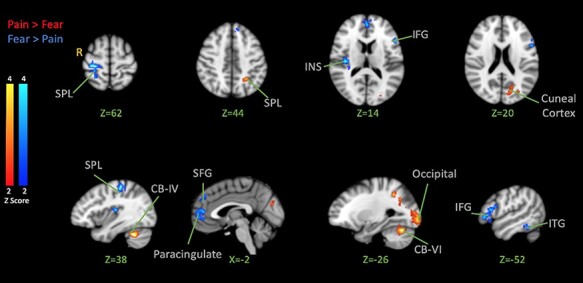
BOLD activation in the brain for Pain > Fear Facial Expression Observation (Red) and Fear > Pain Facial Expression Observation (Blue). The color bar indicates Z-Score. The activation map was cluster-corrected for multiple comparisons using GRF (*P <* 0.01).

Next, we explored whether each negative expression observed would elicit stronger brain responses (Figure 3). Contrasting activation for pain faces *vs* fear faces revealed stronger activation for pain in the left superior parietal lobule, superior part of the lateral occipital cortex, cuneal cortex, occipital pole, right cerebellum V and VI and left cerebellum VI. Stronger activation to fear faces was revealed in the left ITG and middle temporal gyrus (MTG), left IFG, bilateral paracingulate gyrus and ACC, right superior parietal lobule, right post-central gyrus, INS and Heschl’s gyrus.

#### Shock-evoked responses

**Fig. 4.**
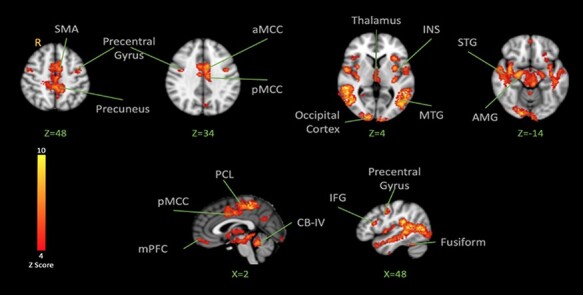
BOLD activation in the brain in response to noxious shocks. The color bar indicates Z-Score. The activation map was cluster-corrected for multiple comparisons using GRF (*P < *0.05). Peak coordinates are reported in Table 2.

After showing how the brain responds to the observation of pain, fear and neutral expressions, we examined how the brain responds to high-intensity shocks across conditions. Analysis of fMRI data of the functional localizer run revealed that the high-intensity shock activated the bilateral INS, bilateral amygdala, bilateral anterior and posterior cingulate cortex, bilateral PCL (with the peak located on the left hemisphere contralateral to the stimulation site), precuneus, bilateral cerebellum I–IV (with the peak located on the right cerebellum), left thalamus, bilateral hippocampus and bilateral operculum (Figure 4 and Table 2). A similar pattern was observed in the main experiment when the high-intensity shocks were applied after the visual stimuli displayed pain, fear or neutral expressions. This confirmed that the high-intensity shocks produced a brain activation pattern generally consistent with previous pain imaging studies (Duerden and Albanese, 2013).

**Table 2.** Brain activation in response to painful stimulation

*Z*-value	*X*	*Y*	*Z*	Anatomical label
12.08	−32	−24	14	Left insula
12.76	60	−42	4	Right middle temporal gyrus
11.92	−10	−36	60	Left precuneus
11.53	−60	−52	5	Left middle temporal gyrus
10.73	−1	−6	34	Anterior cingulate cortex
9.90	52	−32	20	Right superior temporal
9.26	38	−44	−26	Right cerebellum VI
9.08	12	−98	8	Right cuneus
8.96	−38	−46	−28	Left cerebellum VI
7.53	10	−99	2	Right occipital
7.47	−4	2	48	Supplementary motor area
7.26	20	−5	−14	Right amygdala
6.47	50	11	17	Right inferior frontal gyrus
6.35	−2	−16	4	Left thalamus
6.29	48	3	35	Right pre-central gyrus

**Fig. 5.**
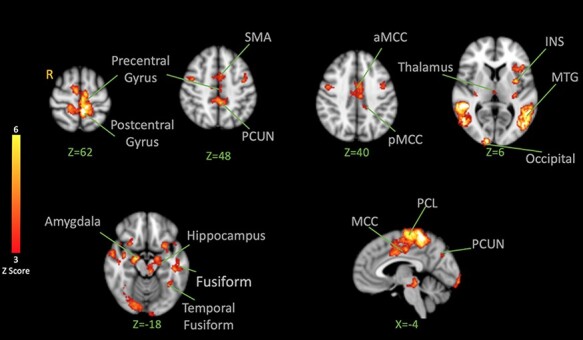
BOLD activation in the brain in response to noxious shocks after observation of pain and fear facial expressions > after observation of neutral facial expressions. The color bar indicates Z-Score. The activation map was cluster-corrected for multiple comparisons using GRF (*P* < 0.05). The strongest peak found in the left INS was used for the PPI analysis. Peak coordinates are reported in Table 3.

Following the motivational priming hypothesis, we then examined the brain’s response to shock following both negative expressions. Contrasting shock-evoked activation following the observation of pain and fear faces together *vs* the activation following the observation of neutral faces resulted in the activation in the post-central gyrus, left INS (anterior, mid and posterior with the highest peak on the left), bilateral thalamus (peak on the right), bilateral MTG, bilateral amygdala, bilateral hippocampus, left temporal occipital fusiform, left fusiform gyrus, MCC, sensorimotor cortices (with the peak on the left side), bilateral precuneus, SMA and right cerebellum (Figure 5; Table 3). These effects reflect significant facilitation of pain responses by one or both negative expressions.

**Table 3.** Brain activation in response to shock following observation of pain and fear facial expressions *vs* neutral expression

*Z*-value	*X*	*Y*	*Z*	Anatomical label
6.21	−8	−40	70	Post-central gyrus
5.87	−35	0	6	Left insula
5.66	50	−44	6	Right middle temporal gyrus
5.43	−58	−56	5	Left middle temporal gyrus
5.28	−10	−68	46	Left precuneus
4.87	18	−7	−18	Right amygdala
4.67	−2	6	40	Anterior cingulate cortex
4.33	12	−68	46	Right precuneus
4.02	24	−32	−6	Right hippocampus
3.89	−2	−3	−48	Supplementary motor area
3.75	−35	−49	−18	Left fusiform
3.68	3	−18	6	Thalamus

**Fig. 6.**
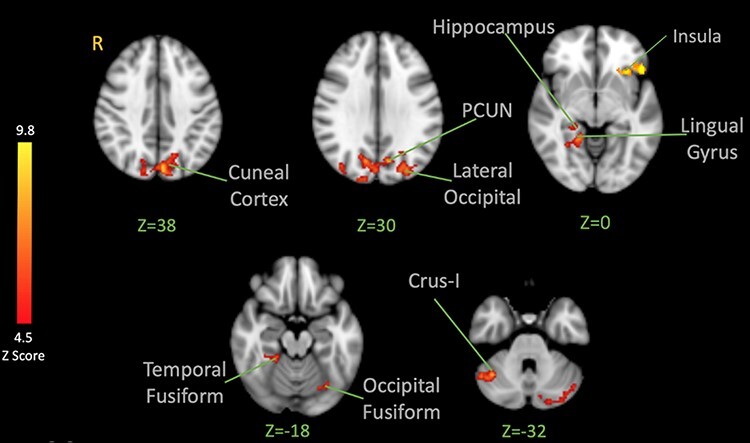
BOLD activation in the brain in response to noxious shocks after observation of pain facial expressions > after observation of fear facial expressions. The color bar indicates Z-Score. The activation map was cluster-corrected for multiple comparisons using GRF (*P* < 0.05). Peak coordinates are reported in Table 4.

On the other hand, we were interested in testing the specificity hypothesis and seeing if/how the brain’s response to painful shocks following the observation of pain expressions might differ from the response following fear. Contrasting shock-evoked activation following the observation of pain faces *vs* fear faces (pain > fear) revealed activation in the left anterior INS (extending into the IFG), bilateral cerebellum crus-I, left cerebellum lobule VI, right lingual gyrus, right posterior hippocampus, temporal occipital fusiform cortex, lateral occipital cortex (both inferior and superior divisions), left cuneal cortex and bilateral precuneus cortex (Figure 6; Table 4).

**Table 4.** Brain activation in response to shock following observation of pain *vs* fear facial expressions

Z-Value	X	Y	Z	Anatomical label
9.83	−30	22	0	Left insula
8.28	−2	−42	58	Left precuneus
8.35	50	−47	−32	Right crus I
7.94	−46	−74	6	Left mid-occipital
7.03	44	−44	−26	Right fusiform
7.04	14	−98	6	Right cuneus
7.04	−42	−44	−24	Left fusiform
4.94	−6	−30	−16	Brain stem

Contrasting shock-evoked activation following the observation of fear faces *vs* pain faces did not reveal significant activation.

#### PPI analysis

**Fig. 7.**
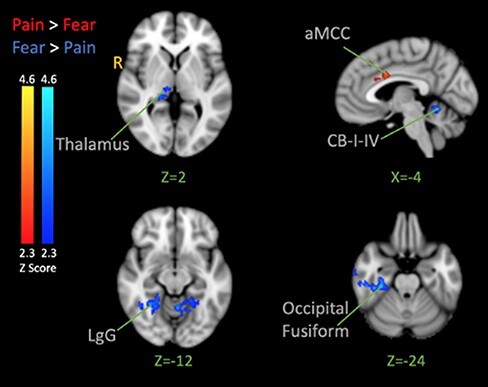
PPI analysis. The seed was the left INS. Contrasts show activation correlated with the activation of the insula moderated by the type of facial expressions that preceded the shock (Pain > Fear is red and Fear > Pain is Blue). The color bar indicates Z-Score. The activation map was cluster-corrected for multiple comparisons using GRF (*P* < 0.05). Peak coordinates are reported in Table 5.

INS is recognized as a multifaceted center involved in the processing and modulation of pain (Starr *et al.*, 2009). The shock-evoked insular response found in the analysis combining the pain and fear face conditions (*vs* neutral) was used as an ROI seed (XYZ = −35, 0, 6, see Table 3). A PPI was then conducted to investigate the INS functional connectivity in response to noxious shocks following the observation of pain and fear *vs* neutral, and pain *vs* fear. When pain and fear were taken together *vs* neutral, the PPI analysis revealed stronger co-activation in the pre-central gyrus, cerebellum, STG, SMG and MTG intracalcarine cortex and lateral occipital cortex (Table S1). The pain alone *vs* neutral contrast resulted in stronger co-activation in the pre-central, right thalamus, bilateral medial orbitofrontal, left ACC, right INS and right pre-central (Table S2). The fear alone *vs* neutral contrast showed activation in the right SPL, right cerebellum IV and V, right lingual gyrus and vermis IV and V (Table S3). Pain *vs* fear contrast revealed stronger co-activation to pain in the left anterior MCC (Figure 7-red; Table 5A). The reverse (fear *vs* pain) resulted in activation in cerebellum lobule I–IV, vermis IV–V, right thalamus, right temporal fusiform gyrus, temporal occipital cortex, MTG and bilateral lingual gyrus (Figure 7 blue; Table 5B).

**Table 5.** Brain activation result of PPI analysis with the seed in the left insula

*Z*-value	*X*	*Y*	*Z*	Anatomical label
A)Pain *vs* Fear				
3.83	−2	8	32	Mid ACC
B)Fear *vs* Pain				
5.24	42	−84	12	Right temporal occipital
4.18	−10	−42	−20	Left cerebellum I–IV
4.06	32	−28	−26	Right fusiform
4.33	23	−40	−12	Right lingual gyrus
3.84	10	−22	2	Right thalamus
3.61	−2	−54	−10	Vermis IV–V

## Discussion

In this study, we showed that observation of pain and fear facial expressions facilitated spinal processing of nociceptive signals, measured by the NFR, and increased pain ratings following a noxious shock. Pain ratings following the observation of a fear expression were also significantly higher than ratings following the observation of a pain expression. Contrasting the effect of pain observation with fear observation (with or without shocks) did not result in stronger activation in areas that are part of the commonly known pain network. These results provide support for the motivational priming account (Lang, 1995) as they suggest that the facilitation of responses to pain is not specific to the observation of pain. The observation of pain or fear expressions can result in the activation of a network in the observer’s brain that leads to elevated pain perception. However, the PPI analysis suggests some differential changes in connectivity, which may reflect the activation of distinct pain modulatory pathways following the observation of pain and fear faces.

### Brain response to the observation of emotional expressions

The brain responses to emotional expression (without a shock) were similar to those reported in many previous studies and meta-analyses (Simon *et al.*, 2006; Jauniaux *et al.*, 2019). When contrasting fear and pain against neutral expressions, we observed activation in the MCC, an area suggested to be involved in the processing of general negative stimuli like pain and disgust in previous studies (Benuzzi *et al.*, 2008). A stronger response to fear and pain expressions was also found in the pre-central gyrus, precuneus, ventral premotor cortex, bilateral MTG and fusiform gyrus, areas previously involved in the processing of dynamic emotional expressions (Sato *et al.*, 2004). Results also showed activation in the IFG, an area suggested to be involved in the interpretation of emotional facial expressions (Budell *et al.*, 2010, 2015). Contrasting pain *vs* fear expressions revealed activation in posterior brain areas such as the cuneal cortex, which is responsive to negative emotion (Kumfor *et al.*, 2013), as well as in the SPL and cerebellum—areas reported to be engaged in action preparation (Schraa-Tam *et al.*, 2012; Engelen *et al.*, 2018). Contrasting fear *vs* pain revealed activation of the ITG and INS, which are known to be interconnected to the amygdala (Aggleton *et al.*, 1980) and regions like the IFG, which are known to be responsive to threat-induced anxiety (Gold *et al.*, 2015). These findings are consistent with distributed brain responses to emotional facial expressions, with pain and fear expressions being associated with partly dissociable networks.

### Brain responses to pain

Brain responses to pain stimulations were highly consistent with previous studies (Duerden and Albanese, 2013). When pain and fear observations were considered together, the contrast *vs* neutral expression revealed stronger shock-evoked activation in the network involved in the processing of sensory and pain information (Wiech and Tracey, 2009), including the PCL, aMCC, INS and the amygdala. Contrasting the brain response to shock following the observation of pain or fear *vs* neutral showed recruitment of similar networks in the brain involving areas such as the INS and MTG, which implies that those shock-evoked responses were stronger following pain and fear faces. When a direct contrast comparing the brain response to shock following pain observation *vs* fear observation was made, no significant activation was observed in the areas traditionally known to be a part of the network involved in the processing of pain. This finding may suggest that observation of negative emotion primes harm-avoiding behavior by facilitation of internal processing of the pain-related signal, which cannot be explained simply by a sensorimotor resonance process that is specific to pain.

Many previous studies showed the facilitation of responses to pain following observation of pain or emotional stimuli (Rhudy *et al.*, 2005; Roy *et al.*, 2009; Vachon-Presseau *et al.*, 2011, 2012; Bayet *et al.*, 2014; Khatibi *et al.*, 2014). This evidence has been built on a body of literature suggesting an overlap between the vicarious experience of pain and the firsthand experience of pain (Morrison *et al.*, 2004). It has been proposed that the observed facilitation is the result of a sensorimotor resonance between the firsthand and vicarious experience of pain. However, some studies have shown the facilitation of responses to pain by observing other types of negatively valenced facial expressions (e.g. sad expression; Bayet *et al.*, 2014). Similarly, some neuroimaging studies investigating the neural mechanisms of compassionate hyperalgesia suggest a partially overlapped pattern of activation in the brain to pain and other negative emotions that questions the sensorimotor resonance hypothesis (Godinho *et al.*, 2012; Krishnan *et al.*, 2016).

Some researchers have suggested that arousal may explain the effect of vicarious pain facilitation (Bernhardt and Singer, 2012; Nazarewicz *et al.*, 2015). These studies propose that the urge for avoidance following the exposure to negative (and potentially threatening) stimuli is a protective regulatory mechanism that helps the person reduce distress following the perception of threat (Nazarewicz *et al.*, 2015). In this study, we included two categories of negative expressions: pain and fear. We also matched the level of arousal in both categories. In our study, pain ratings following observation of fear were higher than following observation of pain or neutral faces. Although arousal was matched between pain and fear expressions, the intensity rating for fear expression was higher than matched pain expressions. Previous studies also suggested that the valence of the expression influences pain ratings, and this may explain the pattern in our findings (Bayet *et al.*, 2014). Higher expression intensity may explain higher pain ratings following observation of fear than pain. However, this needs to be tested independently in future studies. Observation of fear, similar to the observation of pain expression, can send a signal about the existence of a threat in the environment (Zhao *et al.*, 2017). Perception of threat, even under sub-optimal conditions, can result in the facilitation of the observer’s response and can increase arousal (Khatibi *et al.*, 2015). An increase in the arousal for negative valenced experiences is suggested to be associated with an increase in the perception of self-pain (Rainville *et al.*, 2005). Results of the contrast between the brain response to shock following pain and fear expressions did not reveal activation in the areas traditionally known to be involved in the processing of pain, with a possible exception in the most anterior part of the left INS (Figure 6). However, when pain and fear expressions were pooled together, the brain response to the shock following negative expression *vs* neutral expressions involved areas such as the PCL (putative foot area in the primary sensorimotor cortex), MCC, amygdala and INS that have shown in the previous studies to be involved in the emotional modulation of pain (Roy *et al.*, 2009; Godinho *et al.*, 2012; Bushnell *et al.*, 2013). The emotional mechanisms that are activated in response to the observed stimuli may play an important role in the modulation of pain responses.

Understanding shared and specific networks that contribute to the modulation of pain is the key to testing the sensorimotor resonance hypothesis *vs* the motivational priming theory. In the brain response to shock following the observation of pain and fear *vs* the observation of neutral expressions, a number of regions, such as the INS, were active in both conditions. Previous studies investigating the emotional modulation of painful experiences have proposed the involvement of the INS, as this region is proposed to integrate information about the emotional state of the person and interoceptive information (Critchley and Garfinkel, 2017). To further test the specificity hypothesis, we investigated brain networks that co-activate with the INS in those conditions. Following the observation of pain expressions *vs* fear, only one cluster of co-activation was observed in the anterior part of the MCC. The contribution of the ACC and aMCC to both the firsthand and observational experiences of pain has been widely discussed in the literature (Jackson *et al.*, 2006; Lamm *et al.*, 2011; Lieberman and Eisenberger, 2015; Krishnan *et al.*, 2016; Zaki *et al.*, 2016; Zhou *et al.*, 2020). Previous studies suggest that INS (especially the anterior region) co-activates with the ACC (and the anterior portion of MCC) during empathy for pain (Gu *et al.*, 2010). Our results are in line with these previous findings. When comparing negative stimuli (pain and fear) to neutral expressions, our results are in line with the study by Roy *et al.* (2009), as we observe a connection between the activation in the INS and the parahippocampal gyrus (PHG, as a possible result of anticipation of pain and related anxiety), the PCL and the cuneal cortex. This finding is in line with the involvement of the INS in the integration of sensory and emotional information (Craig, 2009). Furthermore, the activations in the fusiform gyrus, MTG and SMG are in line with suggestions regarding their involvement in the processing of emotional expressions and empathic response to distress in others (Lamm *et al.*, 2011). These findings suggest partly distinct functional networks contributing to the vicarious emotional modulation of pain following observation of pain and fear.

### Limitations

Our study has limitations that should be considered when interpreting the results. First, the electrical stimuli were always delivered immediately after the faces, leading to multicollinearity in our design matrix and making it difficult to clearly separate the brain activity evoked by the expressions, the painful stimuli or their interaction. This design can potentially influence the brain response to the observation of facial expressions as they could be seen as a cue for the shock. This effect was balanced across the presentation of different types of expression, but we cannot formally exclude a possible interaction reflecting a differential effect of pain and fear faces on self-pain anticipation. Furthermore, our analysis model did not include a regressor for the face stimulus preceding the noxious shock when examining the modulation of the pain response by facial expressions (see section Brain fMRI analysis). The logic of this approach was to obtain separate maps for (i) face-evoked brain activation unconfounded with the noxious stimulus (i.e. face-only trials; Figure 2), (ii) nociceptive pain evoked by noxious electrical stimuli only (pain functional localizer, Figure 4) and (iii) pain-evoked maps contrasting the three face conditions (Figures 5 and 6). A corollary analysis looking at the modulation of pain-evoked responses in a model including the three face regressors for every trial (i.e. with or without painful shock) yielded few results. This implies that the results reported here reflect the effect of faces on pain responses, the effects of pain or pain anticipation on face processing or a combination of both. Electrophysiological methods with better temporal resolution compared to fMRI might be indicated to solve this issue. Second, we only contrasted pain, fear and neutral expressions using a limited number of validated facial stimuli. A more extensive stimuli set, including more diverse models and richer sets of expressions, would be highly relevant to ensure the generalizability of findings. Third, the sample size was smaller than in similar studies that investigated vicarious pain facilitation. As the effect size estimates suggest, the sample might be big enough for the visualization of the difference in pain ratings following the observation of pain and fear but for NFR, we need a bigger sample to make a comprehensive conclusion. Besides, despite a trend toward significance in the correlation between RIII and pain ratings following the observation of pain and fear, we did not find an association between changes in the rating or RIII following pain–neutral and fear–neutral. Future studies with a bigger sample can help us understand these effects better.

### Conclusion

Taken together, our study generally suggests that the vicarious facilitation of responses to pain is not strictly specific to the observation of pain expressions. Our findings support the effect of negative emotions on the descending facilitation of responses that are activated by the perception of a threat in the environment. Brain regions involved in the observational modulation of pain include INS, aMCC, SMA, sensorimotor cortices, thalamus and PHG. However, the stronger pain responses in the anterior part of the left INS following pain faces combined with the result of the PPI analysis suggest that different networks may contribute more specifically to the emotional modulation of pain when observing pain or fear. Future studies are encouraged to take a closer look at disentangling the effect of arousal and valence on the activation of these descending modulatory mechanisms.

## Supplementary Material

nsac056_Supp
