# Supplementary material for: Brain responses to the vicarious facilitation of pain by facial expressions of pain and fear
Source: Soc Cogn Affect Neurosci. 2022 Oct 6;18(1):nsac056. doi: 10.1093/scan/nsac056 (PMC9949570; doi:10.1093/scan/nsac056)
Supplement: nsac056_Supp [file nsac056_supp.zip › scan-22-072-File014.docx]

| **Table S1: Brain activation result of PPI analysis with the seed in the left insula for Pain and Fear vs Neutral contrast** | | | | |
| --- | --- | --- | --- | --- |
| **Z-Value** | **X** | **Y** | **Z** | **Anatomical Label** |
| 4.45 | 28 | -32 | -26 | Right Cerebellum IV-V |
| 4.60 | 59 | -21 | 36 | Right Supramarginal Gyrus (SMG) |
| 4.50 | 44 | -52 | 10 | Right Mid Temporal (MTG) |
| 3.15 | 38 | -18 | 42 | Right Precentral |
| 2.48 | 4 | -64 | 14 | Right Calcarine |
| 2.35 | 64 | -30 | 14 | Right Superior Temporal Gyrus (STG) |

| **Table S2: Brain activation result of PPI analysis with the seed in the left insula for Pain vs Neutral contrast** | | | | |
| --- | --- | --- | --- | --- |
| **Z-Value** | **X** | **Y** | **Z** | **Anatomical Label** |
| 4.96 | 2 | 36 | 12 | ACC |
| 4.10 | 46 | -16 | 54 | Right Precentral |
| 3.70 | 16 | -6 | 0 | Right Thalamus |
| 3.54 | 8 | 44 | -12 | Right Medial Orbitofrontal |
| 2.64 | 34 | 12 | 12 | Right Insula |
| 2.46 | -2 | 48 | -6 | Medial Orbitofrontal |
| 2.35 | 18 | -24 | 76 | Right Precentral |

| **Table S3: Brain activation result of PPI analysis with the seed in the left insula for Fear vs Neutral contrast** | | | | |
| --- | --- | --- | --- | --- |
| **Z-Value** | **X** | **Y** | **Z** | **Anatomical Label** |
| 5.20 | 26 | -32 | -26 | Right Cerebellum IV-V |
| 4.07 | 22 | -50 | -6 | Right Lingual |
| 3.67 | 16 | -62 | 62 | Right Superior Parietal |
| 2.69 | 6 | -42 | -4 | Vermis IV-V |
